# Effects of *Eisenia bicyclis* Extract on Sleep Promotion in a Caffeine-Induced Insomnia Models

**DOI:** 10.4014/jmb.2509.09008

**Published:** 2025-10-27

**Authors:** Yeon Ji Ha, Sekyung Lee, Hyung Joo Suh, Yejin Ahn, Hyunjae Kim, Yu-Kyong Shin, Ki-Bae Hong

**Affiliations:** 1 Department of Food Science and Nutrition, Jeju National University, Jeju 63243, Republic of Korea; 2 Department of Integrated Biomedical and Life Science, Graduate School, Korea University, Seoul 02841, Republic of Korea; 3 Transdisciplinary Major in Learning Health Systems, Graduate School, Korea University, Seoul 02841, Republic of Korea; 4 Research Group of Functional Food Materials, Korea Food Research Institute, Wanju-gun 55365, Republic of Korea; 5 R&I Center, COSMAX BIO Co., Ltd., Seongnam 13486, Republic of Korea

**Keywords:** Animal, *Eisenia bicyclis*, caffeine, insomnia, sleep

## Abstract

This study investigated the sleep-promoting effects of the brown alga *Eisenia bicyclis* (EB), which contains phlorotannins, using caffeine-induced insomnia models of *Drosophila melanogaster* and ICR mice. In flies exposed to caffeine, EB treatment dose-dependently normalized disrupted nighttime activity and total sleep duration, while high-dose EB significantly reduced sleep fragmentation by decreasing the number of sleep bouts. Locomotor tracking analysis further showed that EB attenuated caffeine-induced hyperactivity, reducing distance moved, velocity, and mobility to levels comparable with the normal and benzodiazepine (BDZ)-treated groups. In the pentobarbital-induced sleep test with mice, EB restored the caffeine-induced reduction in sleep duration, although sleep latency remained unaffected. Moreover, EB significantly reduced elevated brain malondialdehyde levels induced by caffeine, accompanied by increased expression of antioxidant-related enzymes. Neurochemical analyses revealed that EB enhanced the levels of γ-aminobutyric acid (GABA) and serotonin, as well as the expression of their receptors, effectively reversing caffeine-induced reductions. These findings suggest that EB exerts sleep-promoting effects by modulating behavioral activity, enhancing antioxidant defense, and regulating GABAergic and serotonergic neurotransmission. Collectively, our results support the potential application of EB as a marine algae-derived functional material with relevance for both the food and pharmaceutical industries in the management of sleep disorders.

## Introduction

Sleep has a complex and bidirectional relationship with diseases, serving both as a consequence and a risk factor, and numerous sleep disorders are influenced by physical, psychological, and environmental determinants. In addition, unhealthy lifestyle behaviors such as overeating, nighttime eating, alcohol consumption, and caffeine intake have been associated with poor perceived sleep quality. Among sleep disorders, insomnia presents with varying symptoms depending on sex, age, and work schedule [1, 2]. Recent studies have shown that sleep is associated with muscle and organ aging, increased anxiety, the development of depression, and a heightened risk of physical and mental impairments [3-5]. Although pharmacological interventions such as benzodiazepines (BDZ) and zolpidem are commonly employed in the treatment of both short- and long-term insomnia and provide immediate symptom relief, prolonged use is associated with various adverse effects, and abrupt discontinuation can lead to rebound insomnia and withdrawal symptoms

Among the various strategies for alleviating and treating symptoms of insomnia, food-derived approaches targeting inhibitory neurotransmission are considered a suitable option for meeting the growing demand for quality-of-life–oriented interventions. Gamma-aminobutyric acid (GABA), an inhibitory neurotransmitter involved in sleep regulation and associated with anti-hypertensive and antidepressant activities, is the end product of glutamic acid decarboxylation and can be produced by lactic acid bacteria via glutamic acid decarboxylases, enabling its application as a food additive or functional food supplement [6]. Active constituents, including valerenic acid, xanthohumol and apigenin, obtained from food plant extracts, have been shown to bind to ionotropic GABA_A_ receptors to regulate stress reduction and sleep promotion [7]. While recent evidence indicates that tyrosine- and tryptophan-based peptides can also alleviate anxiety and depression via interactions with GABA_A_ receptors as well as modulation of tryptophan metabolism and regulation of the hypothalamic–pituitary–adrenal axis [8, 9].

Polyphenols derived from brown algae have been reported to exert hypnotic effects. In particular, dieckol and triphlorethol A, phlorotannins identified in *Ecklonia cava*, have been shown to improve sleep quality, potentially through modulatory interactions with the GABA_A_ - benzodiazepine receptor [10, 11]. In the case of *Eisenia bicyclis* (EB), hydrophilic interaction chromatography has been employed to examine seasonal variation and extraction characteristics, and the contents of five major phlorotannins such as eckol, phlorofucofuroeckol-A, dieckol, 6,6'-bieckol, and 8,8'-bieckol have been reported [12]. Nevertheless, although considerable attention has been directed toward the bioactive constituents of EB, the sleep-promoting effects of EB extracts have not yet been elucidated in scientific literature.

Caffeine acts as a competitive antagonist at adenosine receptors, producing stimulant effects that contribute to arousal and thereby influence the initiation, fragmentation, and architecture of sleep [13, 14]. The present study aimed to demonstrate the sleep-promoting effects of EB extract by employing both invertebrate and vertebrate models. Specifically, caffeine was used to induce arousal and suppress sleep, and subsequent EB extract treatment was evaluated for the ability to attenuate arousal and enhance sleep. Based on previous findings on behavioral outcomes, sleep duration, antioxidant activity, and target receptor expression, the present study aimed to investigate the potential of EB extract as a candidate functional ingredient for the development of sleep-related nutraceuticals and pharmaceuticals.

## Materials and Methods

### Materials

A 30% ethanol extract of *Eisenia bicyclis* (EB) was provided by COSMAX BIO Co., Ltd. (Republic of Korea). Pentobarbital (Entobar, 100 mg) was purchased from Hanlim Pharmaceutical Co. (Republic of Korea), and alprazolam (Xanax, 0.5 mg; Pfizer Korea Ltd., Republic of Korea) was used as a BDZ reference drug. Unless otherwise specified, all other chemicals and reagents were obtained from Sigma-Aldrich (USA).

### Experimental Animals

This study employed fruit flies and mice as experimental models. Wild-type *Drosophila melanogaster* (Canton-S strain) was obtained from the Drosophila Stock Center at Indiana University (USA). To synchronize circadian rhythms, flies were maintained in an incubator (HB302L, Hanbaek Scientific Co., Republic of Korea) under a 12:12 light-dark cycle. They were reared on a standard diet at 23 ± 1°C and 60–70% relative humidity. The diet consisted of 5.0% sucrose, 2.0% agar, 4.28% cornmeal, 6.8% dried yeast, p-hydroxybenzoic acid methyl ester, and propionic acid. Three-day-old male flies were anesthetized with CO_2_ prior to experimentation. Based on previous studies, insomnia was induced by administering 0.1% caffeine as an arousal-inducing agent, and flies were exposed to a standard diet supplemented with EB extract [15]. Three-week-old male Institute for Cancer Research (ICR) mice were purchased from Samtako Bio Korea (Republic of Korea). All animals were acclimated for 7 days prior to experimentation to minimize stress from environmental adaptation. They were housed under controlled conditions at 24 ± 1°C and 55–60% relative humidity, with a 12 h light/12 h dark cycle. Food and water were freely available throughout the study. All animal procedures were approved by the Institutional Animal Care and Use Committee of Jeju National University (IACUC Approval No. 2024-0041) and conducted in accordance with institutional and national guidelines for the care and use of laboratory animals.

### Assessment of Sleep and Locomotor Activity in *Drosophila melanogaster*

Sleep-associated activity in individual flies was assessed using the *Drosophila* Activity Monitoring (DAM) system (TriKinetics, USA). Each fly was placed in a transparent glass vial (65 × 55 mm), and locomotor activity was recorded continuously at 1-min intervals using an infrared beam positioned at the center of the vial. To assess whether circadian rhythms persisted without external light cues, flies were maintained under constant darkness. They were cultured for 4–6 days at 23 ± 1°C with 60–70% relative humidity and provided with a sucrose–agar medium containing the test material (5% sucrose, 3.5% agar). Sleep was defined as immobility lasting ≥5 min. Sleep-related parameters, including subjective nighttime activity, total sleep duration, and number of sleep bouts, were analyzed. For circadian rhythm analysis, subjective daytime (10:01–22:00) and subjective nighttime (22:01–10:00) were distinguished, and actograms were generated using Actogram J software (version 1.51). To evaluate locomotor behavior, male flies were reared on a sucrose–agar medium supplemented with EB extract for 7 days. After treatment, individual flies were placed in one of nine arenas (8 mm in diameter, 0.1 mm in height). Following a 1-min acclimation period, locomotor activity was recorded for 5 min using a video-based tracking system. Behavioral parameters—including total distance moved, mean velocity, percentage of time spent moving, percentage of time spent immobile, and overall mobility were quantified using the EthoVision XT system (Noldus Information Technology, The Netherlands). Total distance moved was defined as the cumulative path length during the 5-min recording session. Velocity represented the mean displacement rate and was used as an indicator of general activity. Thresholds of 0.2 cm/s and 0.01 cm/s were applied to classify locomotor states as active (moving) or inactive (not moving), respectively. Mobility was expressed as the percentage of time spent in active motion within the arena.

### Pentobarbital-Induced Sleep Test

The pentobarbital-induced sleep test was conducted to evaluate the sleep-promoting effects of EB extract in caffeine-induced sleepless mice. The experimental groups consisted of the normal control (NOR), caffeine-treated control (CON; 50 mg/kg caffeine), positive control (BDZ; 0.2 mg/kg BDZ), low-dose extract (EBL; 125 mg/kg), and high-dose extract (EBH; 250 mg/kg). With the exception of the NOR group, all animals received caffeine treatment. After a one-week acclimatization period, the designated treatments were administered orally once daily for seven consecutive days. Before the final experiment, mice were fasted for 24 h. On the test day, the respective treatment was administered orally, and 40 min later, pentobarbital (42 mg/kg) was injected intraperitoneally. The animals were then placed individually in separate cages for behavioral monitoring. Sleep onset was determined by the loss of the righting reflex. Sleep latency was defined as the time from pentobarbital injection to the loss of the reflex, whereas sleep duration was defined as the interval between the loss and recovery of the reflex. Animals failing to exhibit sleep onset within 15 min after pentobarbital injection were considered non-responders and excluded from subsequent analyses.

### Measurement of Oxidative Stress Biomarkers

Oxidative tissue damage was evaluated by quantifying reactive oxygen species (ROS) and malondialdehyde (MDA), which are widely recognized biomarkers of oxidative stress. Brain tissues were homogenized in 0.01 M phosphate-buffered saline (PBS, pH 7.4) and centrifuged at 10,000 ×*g* for 15 min at 4°C. The resulting supernatants were collected for subsequent analyses. ROS levels were measured using a fluorometric method with a commercial ROS Assay Kit (Invitrogen, USA), while MDA levels were determined with the OxiTec™ TBARS Assay Kit (BIOMAX, Republic of Korea).

### Quantitative Real-Time Polymerase Chain Reaction Analysis

ICR male mice were orally administered *Eisenia bicyclis* extract at doses of 125 or 250 mg/kg once daily for seven consecutive days. After CO_2_ anesthesia, the animals were euthanized, and brain tissues were rapidly excised for molecular analyses. Total RNA was isolated from the collected tissues using RNAzol RT reagent (Sigma-Aldrich) according to the manufacturer’s instructions. To prevent genomic DNA contamination, the extracted RNA was treated with RQ1 RNase-free DNase (Promega, USA). Complementary DNA was synthesized from purified RNA using SuperScript III Reverse Transcriptase (Invitrogen) with oligo(dT) primers. Quantitative real-time PCR (qRT-PCR) was conducted with the TaqMan PCR Master Mix (Applied Biosystems, USA) on a StepOnePlus Real-Time PCR System (Applied Biosystems). Relative gene expression levels were determined by the comparative Ct (ΔΔCt) method. Glyceraldehyde-3-phosphate dehydrogenase (GAPDH, assay ID: Mm0180221_g1) was used as the endogenous control for normalization. The following genes were analyzed in mouse brain tissue: GABA_A_ receptor (NM_008076.3), GABA_B_ receptor subunit 1 (NM_019439.3), GABA_B_ receptor subunit 2 (NM_001081141.1), 5-hydroxytryptamine receptor 1A (Htr1a, NM_008308.4), superoxide dismutase (SOD, NM_011434.2), catalase (CAT, NM_009804.2), and glutathione peroxidase 1 (GPx1, NM_001329528.1).

### Enzyme-Linked Immunosorbent Assay (ELISA) for Neurotransmitter Contents

To investigate the effect of EB extract on inhibitory neurotransmitters such as GABA and 5-hydroxytryptamine (5-HT), ELISA were performed on mouse brain tissues. All treatments were administered orally once daily for seven consecutive days. Following CO_2_ anesthesia, animals were euthanized, and brain tissues were promptly collected. The harvested tissues were homogenized in PBS and centrifuged at 5,000 ×*g* for 15 min at 4°C. Supernatants were analyzed using a Mouse GABA ELISA Kit (Cat# MBS725233) and a Mouse 5-HT ELISA Kit (Cat# MBS1601042; MyBioSource Inc., USA), according to the manufacturers’ instructions. Absorbance was measured at the recommended wavelength according to the manufacturer’s protocol using a microplate reader (Tecan Trading AG, Switzerland). Total protein was quantified using a BCA Protein Assay Kit (Thermo Fisher Scientific), and neurotransmitter concentrations were normalized to protein content and expressed as nanograms per milligram of protein (ng/mg).

### Statistical Analysis

All data are expressed as the mean ± standard error of the mean (SEM). Statistical significance was set at *p* < 0.05. Comparisons between two groups were performed using Student’s *t*-test, whereas differences among multiple groups were analyzed by one-way ANOVA followed by Tukey’s post hoc test. Statistical analyses were carried out using SPSS software (version 24.0; SPSS Inc., USA).

## Results

### Effects of EB Extract on Sleep-Related Parameters in Caffeine-induced Fruit Fly Model of Insomnia

**Fig. 1:**
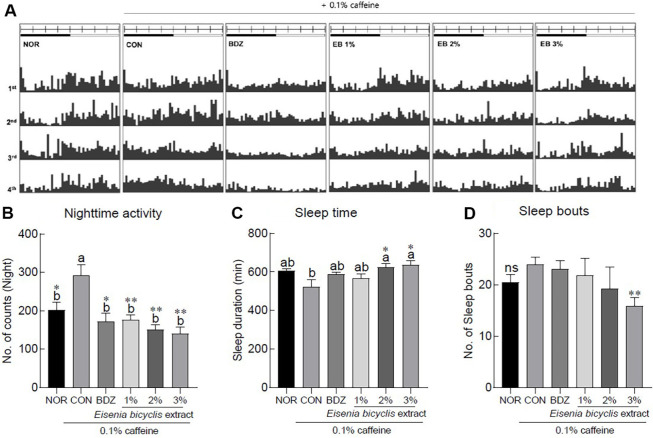
Effects of *Eisenia bicyclis* (EB) extract on sleep-related activity in caffeine-induced *Drosophila melanogaster* insomnia model. (**A**) Typical actograms of individual normal flies and flies exposed to caffeine and/or EB extract. (**B**) subjective nighttime activity. (**C**) subjective nighttime sleep duration. (**D**) number of sleep episodes. Data are presented as the mean ± the standard error of mean (SEM) for each group. Different letters indicate significant differences at *p* < 0.05 using Tukey’s multiple range test. *p < 0.05, **p < 0.01, and ***p < 0.001 vs CON using Student’s *t*-test. NOR, normal group; CON, caffeine-induced insomnia group.

Exposure to caffeine significantly increased subjective nighttime locomotor activity by approximately 1.44-fold in the CON group compared to the NOR group, whereas treatment with BDZ or EB extracts significantly attenuated the arousal effects induced by caffeine (Fig. 1B, *p* < 0.05). Compared to the NOR group, caffeine exposure tended to reduce total sleep time in the CON group, while BDZ treatment attenuated the arousal effect of caffeine, restoring sleep duration to a level comparable to that of the NOR group; notably, treatment with 2% and 3% EB extract significantly increased sleep time relative to the CON group (Fig. 1C, *p* < 0.05). The 3% EB extract significantly decreased the number of subjective nighttime sleep episodes compared to the caffeine-exposed CON group, indicating a potential improvement in sleep quality, as a reduction in sleep fragmentation is generally associated with enhanced sleep quality (Fig. 1D, *p* < 0.01).

### Effects of EB Extract on Locomotor Activity in Caffeine-induced Fruit Fly Model of Insomnia

**Fig. 2:**
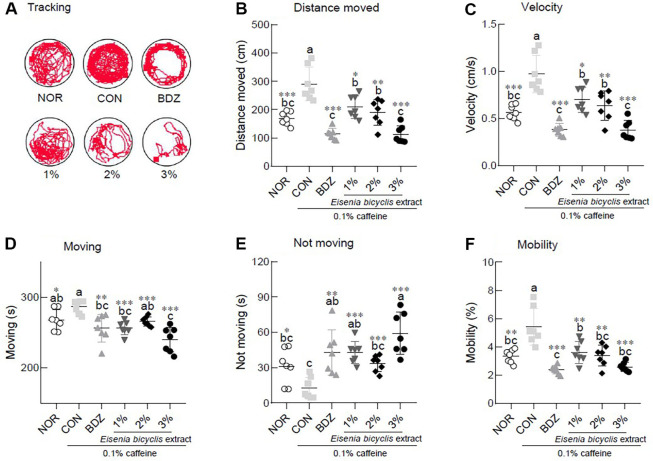
Effects of *Eisenia bicyclis* (EB) extract on locomotor activity in caffeine-induced *Drosophila melanogaster* insomnia model. After 5 days of exposure, the locomotion during the 5 min observation period in the video tracking was analyzed using the EthoVision-XT system. Data are presented as the mean ± the standard error of mean (SEM) for each group. Different letters indicate significant differences at *p* < 0.05 using Tukey’s multiple range test. *p < 0.05, **p < 0.01, and ***p < 0.001 vs CON using Student’s *t*-test. NOR, normal group; CON, caffeine-induced insomnia group.

In the video tracking-based open field test, caffeine exposure (CON group) resulted in a 1.72-fold and 1.70-fold increase in distance moved and velocity compared to the NOR group, whereas treatment with BDZ or EB extract significantly suppressed caffeine-induced arousal (Fig. 2B and 2C). Furthermore, EB extract reduced locomotor activity in a dose-dependent manner, as indicated by a significant decrease in distance moved and velocity compared to the CON group. Caffeine exposure led to significant differences between the NOR and CON groups in both moving and not moving behaviors, and its behavioral effects were significantly modulated by BDZ and EB extract treatments (Fig. 2D and 2E, *p* < 0.05). Moreover, BDZ and high-dose EB extract induced significant alterations in locomotor activity compared with the NOR group (Fig. 2D and 2E, *p* < 0.01 and *p* < 0.001). Mobility increased by approximately 1.63-fold in the CON group compared to the NOR group, reflecting a significant stimulatory effect of caffeine on locomotor activity (Fig. 2F, *p* < 0.01). The elevated mobility caused by caffeine was markedly reduced following treatment with BDZ and EB extract, indicating that both interventions effectively counteracted caffeine-induced hyperactivity (Fig. 2F, *p* < 0.01).

### Effects of EB Extract on Sleep Latency and Duration in Caffeine-induced ICR Mouse Model of Insomnia

**Fig. 3:**
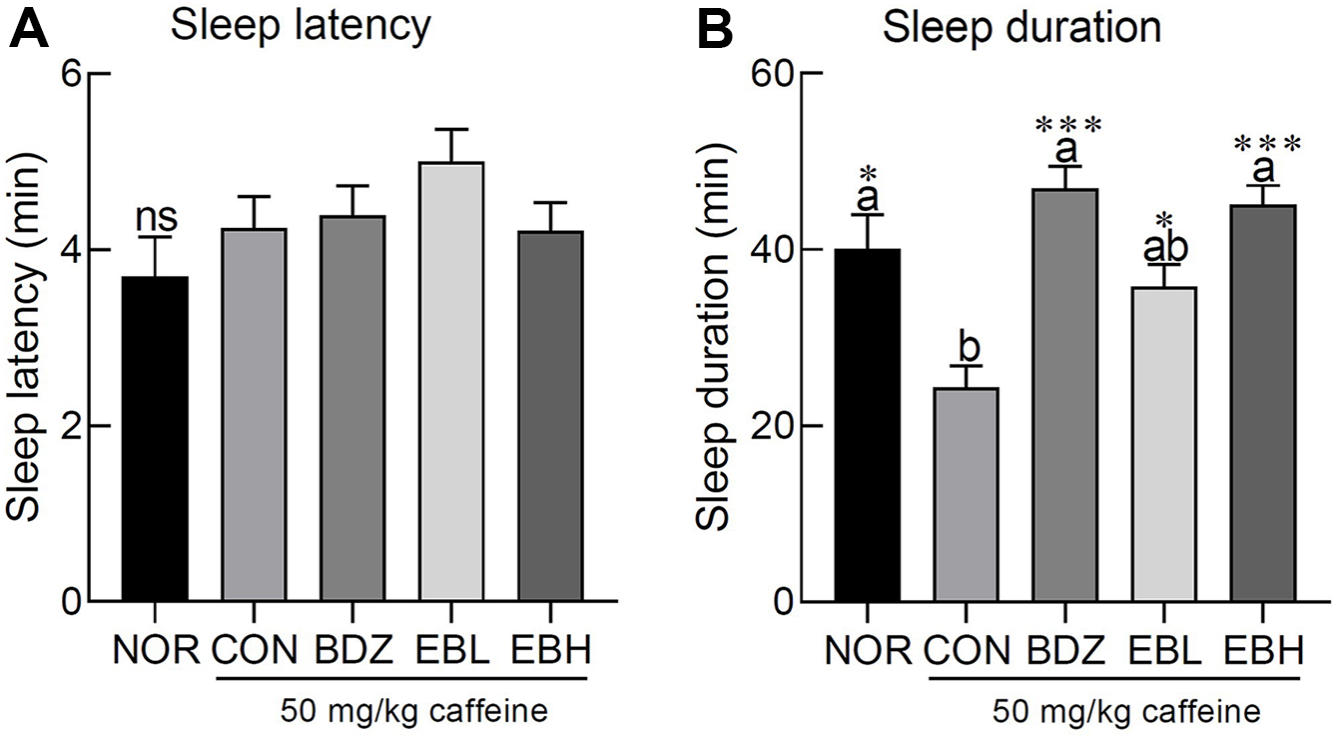
Effects of *Eisenia bicyclis* (EB) extract on (**A**) sleep latency time and (**B**) sleep duration in caffeineinduced ICR mouse insomnia model intraperitoneally injected pentobarbital (42 mg/kg). Values are the means ± standard error of the mean (SEM) for each group. Different letters indicate significant differences at *p* < 0.05 using Tukey’s multiple range test. *p < 0.05, **p < 0.01, and ***p < 0.001 vs CON using Student’s *t*-test. NOR, normal group; CON, caffeineinduced insomnia group 50 mg/kg; benzodiazepine (0.2 mg/kg), BDZ; *Eisenia bicyclis* extract (125 mg/kg), EBL; *Eisenia bicyclis* extract (250 mg/kg), EBH.

Caffeine at a dose of 50 mg/kg did not produce a significant change in sleep latency in ICR mice, and no significant differences were observed between the NOR group and those treated with caffeine in combination with BDZ or either dose of EB extract (Fig. 3A). Sleep duration was reduced by approximately 1.65-fold in the CON group compared to the NOR group following caffeine administration (Fig. 3B, *p* < 0.05). BDZ suppressed the arousal effect induced by caffeine, while also increasing sleep duration beyond the level observed in the NOR group (Fig. 3B, *p* < 0.001). Furthermore, both doses of EB extract (125 and 250 mg/kg) increased sleep duration in a dose-dependent manner, with the higher dose restoring sleep time to levels comparable to those observed in the NOR and BDZ groups (Fig. 3B, *p* < 0.001).

### Effects of EB Extract on Antioxidant Activity in Caffeine-induced ICR Mouse Model of Insomnia

**Fig. 4:**
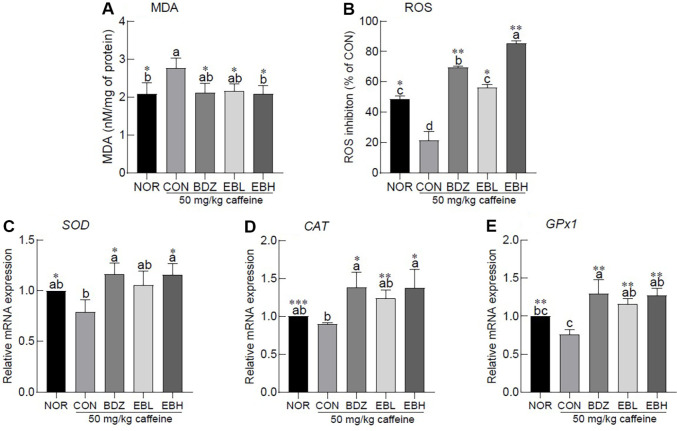
Effects of *Eisenia bicyclis* extract on mRNA expression associated with antioxidant activity of ICR mouse insomnia model. (**A**) malondialdehyde (MDA), (**B**) reactive oxygen species (ROS), (**C**) superoxide dismutase (SOD), (**D**) catalase (CAT), and (**E**) glutathione peroxidase (GPx1). Values are the means ± standard error of the mean (SEM) for each group. Different letters indicate significant differences at *p* < 0.05 using Tukey’s multiple range test. *p < 0.05, **p < 0.01, and ***p < 0.001 vs CON using Student’s *t*-test. NOR, normal group; CON, caffeine-induced insomnia group 50 mg/kg; benzodiazepine (0.2 mg/kg), BDZ; *Eisenia bicyclis* extract (125 mg/kg), EBL; *Eisenia bicyclis* extract (250 mg/kg), EBH.

Caffeine administration markedly increased MDA, a byproduct of lipid peroxidation, in the mouse brain by approximately 1.32-fold relative to the NOR group, indicating a significant elevation of oxidative stress (Fig. 4A, *p* < 0.05). Conversely, treatment with BDZ or EB extract significantly reduced MDA levels compared with the CON group (Fig. 4A, *p* < 0.05). Moreover, ROS scavenging capacity was decreased by approximately 2.28-fold in the CON group relative to the NOR group, whereas administration of BDZ or EB extract significantly restored this activity in a dose-dependent manner (Fig. 4B, *p* < 0.05). The expression of SOD, an antioxidant-related gene, was significantly lower in the CON group than in the NOR group following caffeine administration (Fig. 4C, *p* <0.05). In groups treated with BDZ or high-dose EB extract, SOD expression was significantly elevated relative to the CON group (Fig. 4C, *p* < 0.05). The expression levels of the antioxidant-related genes CAT and GPx1 were significantly reduced in the CON group following caffeine administration compared to the NOR group (Fig. 4D and 4E, *p* < 0.001 and *p* < 0.01). In contrast, groups treated with BDZ or either dose of EB extract exhibited a significant upregulation of both genes relative to the CON group (Fig. 4D and 4E, *p* < 0.05 and *p* < 0.01, respectively).

### Effects of EB Extract on Sleep-Related Neurotransmission in Caffeine-Induced ICR Mouse Model of Insomnia

**Fig. 5:**
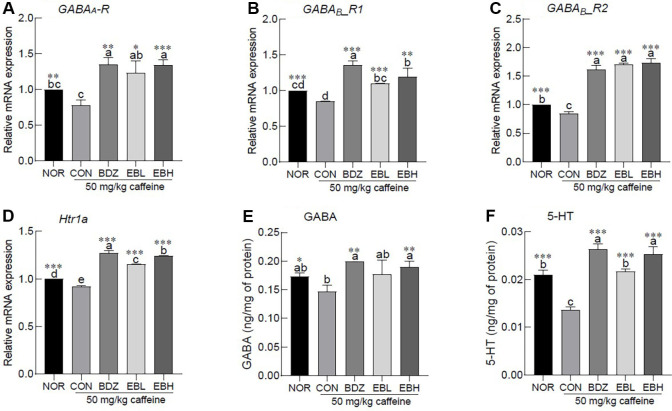
Effects of *Eisenia bicyclis* extract (EB) on mRNA expression associated with sleep of ICR mouse insomnia model. (**A**) GABA_A_-R, (**B**) GABA_B_-R1, (**C**) GABA_B_-R2, (**D**) 5-hydroxytryptamine receptor 1A, (**E**) GABA, and (**F**) 5-HT. Values are the means ± standard error of the mean (SEM) for each group. Different letters indicate significant differences at *p* < 0.05 using Tukey’s multiple range test. *p < 0.05, **p < 0.01, and ***p < 0.001 vs CON using Student’s *t*-test. NOR, normal group; CON, caffeine-induced insomnia group 50 mg/kg; benzodiazepine (0.2 mg/kg), BDZ; *Eisenia bicyclis* extract (125 mg/kg), EBL; *Eisenia bicyclis* extract (250 mg/kg), EBH; gamma-aminobutyric acid, GABA; 5-hydroxytryptamine, 5-HT.

Caffeine administration led to a significant downregulation of brain mRNA expression of the inhibitory neurotransmitter receptors, including the ionotropic receptor GABA_A_-R and the metabotropic receptors GABA_B_-R1 and GABA_B_- R 2 (Fig. 5A, 5B and 5C, *p* < 0.01 and *p* < 0.001, respectively). Co-administration of 50 mg/kg caffeine with either BDZ or EB extract enhanced the expression levels of these target genes (Fig. 5A, 5B and 5C, *p* < 0.05, *p* < 0.01 and *p* < 0.001, respectively). Importantly, treatment with BDZ or high-dose EB extract resulted in significantly higher expression levels than those observed in the NOR group. Caffeine administration was associated with a reduction in the mRNA expression of Htr1a, a G protein-coupled receptor involved in serotonergic signaling (Fig. 5D, *p* < 0.001). Treatment with BDZ or EB extract prevented the caffeine-induced downregulation of Htr1a and led to significantly higher expression levels compared to the NOR group (Fig. 5D, *p* < 0.001). Moreover, EB extract increased Htr1a expression in a dose-dependent manner. Compared to the NOR group, the CON group, which received caffeine alone, exhibited a significant reduction in the levels of the inhibitory neurotransmitter GABA and the sleep-related neurotransmitter 5-HT (Fig. 5E and 5F, *p* < 0.05 and *p* < 0.001, respectively). GABA concentrations in the BDZ-treated and high-dose EB extract groups were restored to levels comparable to those observed in the NOR group. Additionally, EB extract administration resulted in a dose-dependent increase in 5-HT levels relative to the CON group, with the high-dose EB group showing values similar to those of the BDZ group.

## Discussion

Marine brown algae are a rich source of bioactive compounds with diverse physiological functions. Among them, the edible brown alga EB has attracted considerable attention due to its content of fucosterol and six well-characterized phlorotannins. These compounds have been reported to exhibit potent anti-inflammatory, antioxidant, and antidiabetic activities, underscoring the potential of EB as a functional food ingredient [15, 16]. However, despite these well-documented bioactivities, the effects of EB on sleep regulation have not yet been investigated. Caffeine-induced arousal in animal models has been widely recognized as a reliable experimental paradigm for investigating the neurobiological and physiological mechanisms underlying sleep regulation. Such an approach provides a scientifically robust framework for evaluating the sleep-promoting potential of dietary materials and bioactive compounds.

In this study, we evaluated whether exposure to EB extract modulates sleep-related behaviors in a caffeine-induced sleepless *Drosophila* model (Fig. 1). The molecular mechanisms underlying circadian rhythm regulation were elucidated in *Drosophila*, and subsequent studies have reported that dietary neuromodulators such as caffeine and taurine influence sleep–wake cycles by altering neurotransmission and neuronal excitability [17, 18]. Additional evidence further supports the use of *D. melanogaster* as an effective model for investigating sleep through behavioral analyses. For instance, a novel anti-insomnia herbal formula was shown to alleviate caffeine-induced arousal and improve sleep parameters in flies, suggesting its potential application in complementary medicine [19]. Jo *et al*. [20] employed the caffeine-induced *Drosophila* arousal model to examine circadian rhythm and sleep-related behavioral parameters, and demonstrated that extracts of *Nelumbo nucifera* and *Polygonatum sibiricum* possess potential applications as complementary and alternative medicine for sleep improvement.

We also assessed the effect of EB extract on locomotor activity in caffeine-induced arousal *Drosophila* (Fig. 2). The open field test is primarily employed to investigate anxiety-like behaviors or social interactions in *Drosophila* and is not designed to directly assess sleep promotion via neuronal inhibition. Nevertheless, when combined with data obtained from the DAM system, it serves as complementary behavioral evidence for evaluating the efficacy of candidate sleep-modulating dietary materials. In our previous study, the open-field test was employed to determine the optimal mixing ratio of *Ziziphus jujuba*, *Dimocarpus longan*, and *Lactuca sativa*, and the formulation was subsequently shown to promote sleep through modulation of GABAergic signaling [21]. Using mice and rats, the *Camellia sinensis* polyphenol (−)-epigallocatechin-3-O-gallate (EGCG) was shown to reverse caffeine-induced anxiogenic-like behaviors, as demonstrated by the elevated plus-maze and open-field tests, and electroencephalographic analysis further revealed that EGCG attenuated the caffeine-induced increase in the fast wave/slow wave ratio [22].

Our findings indicate that EB extract modulated sleep duration in caffeine-induced insomnia mice, with effects that were comparable to those of BDZ treatment (Fig. 3). Although caffeine is recognized as an arousal-inducing agent, it did not exert an immediate effect on sleep latency, a result consistent with previous findings from short-term administration [23]. In the case of the edible brown seaweed *Ecklonia cava* Kjellman, ethanol extracts and solvent fractions were shown to significantly enhance sleep in a pentobarbital-induced sleep test, and the magnitude of the effect was proportional to the total phlorotannin content, which was reported to be associated with GABA-mediated inhibitory neurotransmission [10]. In addition, chromatographic techniques have revealed that EB extract contains phlorotannins such as eckol and dieckol, which bind to the GABA_A_ -BDZ receptor, as well as other bioactive compounds including fucofuroeckol A, phlorofucofuroeckol A, 8,8'-bieckol, and phloroglucinol [23].

The present findings demonstrated that EB extract modulates brain oxidative stress, which may contribute to its observed sleep-promoting activity (Fig. 4). A bidirectional relationship exists between sleep and oxidative stress, in which the accumulation of reactive species such as ROS and reactive nitrogen species contributes to sleep loss and disturbances [24]. Caffeine is a xanthine alkaloid that stimulates the central nervous system and is widely utilized for lipolytic and performance-enhancing effects; however, sustained arousal in the brain induced by caffeine intake may diminish antioxidant capacity and lead to the accumulation of oxidative stress [25, 26]. Solvent fractions of EB were shown to suppress intracellular ROS and Ca^2+^ generation in PC12 neuronal cells, thereby reducing cell death and exerting neuroprotective effects [27]. Furthermore, active constituents of EB extract, including dieckol and phlorofucofuroeckol A, exhibit strong radical-scavenging activity and suppress oxidative stress, and have also been proposed as potential preservatives for improving food quality [28].

The influence of EB extract on GABAergic and serotonergic pathways was confirmed through mRNA expression analysis and quantification of neurotransmitter levels (Fig. 5). Caffeine has been reported to exert a Ca^2+^-independent inhibitory effect on GABAergic inhibition in the brain [29]. GABA produces both immediate and sustained sedative effects through ionotropic and metabotropic receptors, whereas serotonin serves as a precursor of melatonin and is closely associated with sleep regulation. Phlorotannins from brown algae have been reported to enhance sleep through binding to the GABA_A_ - BDZ receptor, and dieckol contained in edible brown algae has additionally been shown to increase serotonin and norepinephrine levels, indicating its potential application in the treatment of mood disorders [30].

## Conclusion

The present study demonstrated that EB extract exerts significant sleep-promoting effects in both *D. melanogaster* and ICR mouse models of caffeine-induced insomnia. EB treatment restored disrupted sleep parameters, reduced locomotor activity, and improved sleep duration, showing effects that were comparable in trend to those observed with BDZ treatment. These effects were accompanied by attenuation of oxidative stress, upregulation of antioxidant-related enzymes, and enhancement of GABAergic and serotonergic signaling pathways. Collectively, these findings provide preliminary evidence supporting the potential of EB extract as a marine algae-derived functional material for improving sleep and managing sleep disorders in the context of functional food and pharmaceutical applications.
